# Effects of Photobiomodulation on Metabolic, Inflammatory, and Neurological Outcomes in Type 2 Diabetes: A Systematic Review and Meta-Analysis

**DOI:** 10.3390/ijms27010440

**Published:** 2025-12-31

**Authors:** Anne Wevers, Silvia San Roman-Mata, Santiago Navarro-Ledesma, Leo Pruimboom

**Affiliations:** 1 Clinical Medicine and Public Health PhD Program, Faculty of Health Sciences, University of Granada, 18071 Granada, Spain; info@anne-wevers.com; 2 Department of Nursing, Faculty of Health Sciences, Campus of Melilla, University of Granada, 52004 Melilla, Spain; silviasanroman@ugr.es; 3 Research Unit of Excellence of the Melilla University Campus (UECUMEL), Santander Street 1, 52005 Melilla, Spain; 4 Department of Physical Therapy, Faculty of Health Sciences, Campus of Melilla, University of Granada, 52004 Melilla, Spain; 5 Chair in Clinical Psychoneuroimmunology, University of Granada and PNI Europe, Querol Street 5, 54002 Melilla, Spain; leo@cpnieurope.com; 6 PNI EUROPE, 2518 JP The Hague, The Netherlands

**Keywords:** photobiomodulation therapy, type 2 diabetes mellitus, metabolic outcomes, inflammation, neurological outcomes, periodontal outcomes, systematic review, meta-analysis

## Abstract

Type 2 diabetes mellitus (T2DM) is a global health burden characterized by hyperglycemia, oxidative stress, and systemic inflammation, which leads to complications that remain insufficiently managed by standard therapies. Photobiomodulation therapy (PBMT) has been proposed to be a complementary approach, but its effects in T2DM are unclear. We conducted a systematic review and meta-analysis of randomized controlled trials to evaluate the effects of PBMT on metabolic, inflammatory, and neurological outcomes in adults with T2DM. Five databases were searched until June 2025 (PROSPERO CRD420251083550) for relevant studies. Metabolic, inflammatory, and neurological outcomes were defined a priori as primary outcomes and were synthesized narratively due to substantial heterogeneity and incomplete reporting that precluded valid quantitative pooling. Although periodontal outcomes were not predefined as primary outcomes, they were reported in multiple trials; thus, these were analyzed quantitatively as secondary outcomes where sufficient homogeneity enabled meta-analysis. The narrative synthesis of primary outcomes showed inconsistent and largely short-term effects on glycemic control, systemic inflammation, and neurological function. In contrast, meta-analysis of secondary periodontal outcomes demonstrated modest but statistically significant improvements in clinical attachment level (−0.21 mm) and probing depth (−0.25 mm), with no effect on plaque index. Overall, the certainty of the evidence was low. PBMT may offer statistically significant but small adjunctive periodontal effects in adults with T2DM. However, the certainty of evidence is low, and these effects are unlikely to be clinically meaningful in isolation. Evidence for systemic metabolic, inflammatory, and neurological outcomes is preliminary and requires confirmation in larger, standardized RCTs.

## 1. Introduction

T2DM is a significant global health concern, with approximately 830 million adults affected worldwide [1]. This metabolic disorder is characterized by insulin resistance and progressive pancreatic β-cell dysfunction that results in chronic hyperglycemia [2,3]. The prolonged state of hyperglycemia induces excess production of reactive oxygen species, mitochondrial dysfunction, and systemic low-grade inflammation [4]. These processes disrupt cellular metabolism, activate pro-inflammatory signaling cascades, and contribute to cardiovascular, renal, and neurological complications that increase morbidity, disability, and premature mortality [4].

The standard management of T2DM involves positive lifestyle behavioral changes and pharmacological therapy to achieve glycemic control [5]. Nevertheless, these approaches are challenged by poor long-term adherence [6], adverse drug effects [7], and incomplete resolution of oxidative and inflammatory disturbances. Consequently, there is a need to detect complementary therapies that target the underlying biological mechanisms of T2DM, with the aim of delaying or preventing the progress of complications.

PBMT, also known as low level light therapy, is a non-invasive phototherapy that uses red or near-infrared light to stimulate cellular processes [8]. Its primary molecular target is cytochrome c oxidase in the mitochondrial respiratory chain, where photon absorption promotes adenosine triphosphate (ATP) production and modulates redox balance [8]. These changes trigger secondary cellular responses that reduce oxidative stress, regulate inflammatory cytokine production, and support neuronal repair and regeneration [9,10]. Based on these mechanisms, PBMT may provide benefits to the metabolic, inflammatory, and neurological outcomes relevant to T2DM.

Preclinical studies and clinical trials suggest that PBMT may improve glycemic control, reduce inflammatory responses, and alleviate neuropathic symptoms in T2DM [11,12]. However, findings are inconsistent, with some studies reporting only short-term or minimal effects, leading to uncertainty about the clinical significance of PBMT in T2DM treatment. These discrepancies may be attributed to different study designs, PBMT parameters (e.g., wavelength, dose, irradiation site), and different outcome measures; debate over whether its effects are primarily localized or systemic are ongoing. To the best of our knowledge, no synthesis of this evidence has been conducted.

The primary aim of this review was to evaluate the effects of PBMT on metabolic, inflammatory, and neurological outcomes in adults with T2DM. These outcomes were predefined in the registered protocol and reflect the hypothesized systemic mechanisms of PBMT.

During screening, we observed that many trials that reported these outcomes also assessed periodontal parameters. Although they were not prespecified as primary outcomes, these periodontal measures were reported alongside primary outcomes with sufficient statistical detail. Therefore, periodontal outcomes were analysed as secondary outcomes, with quantitative synthesis conducted only where methodological and statistical homogeneity permitted. This approach maintained the primacy of the systemic outcomes while facilitating the meta-analysis of secondary outcomes that met the criteria established for pooling.

## 2. Methods

### 2.1. Protocol and Registration

We conducted this systematic review and meta-analysis by following the Preferred Reporting Items for Systematic Reviews and Meta-analysis (PRISMA) 2020 guidelines [13]. The review protocol was registered in PROSPERO (registration number: CRD420251083550). The methodology was designed to address the research question based on the recommendations provided in *The Cochrane Handbook for Systematic Reviews of Interventions* [14].

### 2.2. Eligibility Criteria

The “Population, Intervention, Comparison, and Outcomes, and Study” (PICOS) framework was applied to select studies that were eligible for inclusion in this systematic review. The inclusion and exclusion criteria based on PICOS framework were as follows:

- Population: Adults diagnosed with T2DM, with or without comorbidities (e.g., peripheral neuropathy). Studies on type 1 diabetes, gestational diabetes, prediabetes, adolescents, animals, or in vitro models were excluded.
- Intervention: PBMT therapy, including low-level laser, light-emitting diode, or infrared light therapy at wavelength ranges between 600 to 1100 nm. Studies where PBMT was applied only for cosmetic purposes, outside of the wavelength ranges, or as a photodynamic therapy were excluded.
- Comparator: Sham PBMT, placebo, usual care, or no intervention. Studies without any control or with unrelated comparators were excluded.
- Outcomes:
  ○ Primary outcomes: At least one metabolic (e.g., glycated hemoglobin, fasting glucose, oral glucose tolerance), inflammatory (e.g., IL-6, TNF-α, C-reactive protein), or neurological outcome (e.g., neuropathic pain, quality of life, nerve conduction, heart rate variability) accounted for. Studies were excluded if the outcome of interest was not measured.
  ○ Secondary outcomes: Periodontal clinical parameters (e.g., clinical attachment level, probing depth, plaque index, bleeding indices, gingival crevicular fluid biomarkers) were reported alongside the primary outcomes.
- Study design: Randomized controlled trials (RCTs) (parallel, crossover, or split-mouth), published in English as full-text, peer-reviewed articles without restrictions on the publication year were included. Non-randomized designs, conference abstracts, reviews, letters, comments, clinical trial protocols and non-English studies were excluded.

### 2.3. Information Sources and Search Strategy

We conducted a literature search in five databases: (1) MEDLINE; (2) Scopus; (3) Web of Science; (4) Cochrane Library; and (5) CINAHL from inception to 20 June 2025. No date restrictions were applied. The search was limited to English language, and peer-reviewed publications according to the eligibility criteria.

The search strategies were initially based on the protocol registered in PROSPERO, which included the free-text terms. However, to minimize the risk of missing relevant studies, we expanded the strategies to include controlled vocabulary (e.g., MeSH, Emtree, CINAHL headings) with free-text keywords. Boolean operators (AND/OR) and database syntax were applied to detect terms related to PBMT, T2DM, and metabolic, inflammatory, and neurological outcomes.

The full search strategies applied for each database, including all controlled vocabulary, keywords, and syntax are provided in Appendix A, Table A1.

### 2.4. Study Selection and Data Collection

All retrieved publications were imported in Rayyan, an online systematic review management platform [15]. Two reviewers (A.W. and C.D.M.S.) independently screened titles and abstracts, followed by full-text assessment against the eligibility criteria. Disagreements were resolved via discussions between the two reviewers, with a third reviewer (S.N.L.) acting as arbiter when consensus could not be reached.

The literature search obtained a total of 332 records from five databases: MEDLINE (*n* = 53), Scopus (*n* = 131), Web of Science (*n* = 65), Cochrane Library (*n* = 63), and CINAHL (*n* = 20). After removal of 98 duplicates, 234 records remained for title and abstract screening. Of these, 190 were excluded for not meeting the inclusion criteria (e.g., irrelevant study design, population, intervention, or outcomes). Of the 44 potentially eligible studies, 41 full texts were successfully retrieved and assessed for eligibility, while 3 could not be obtained.

After full-text screening, 31 articles were excluded, mainly because the reported outcomes were outside the scope of this review (*n* = 16); they involved non-T2DM or animal populations (*n* = 9), or evaluated interventions other than PBMT (*n* = 6).

A total of 10 studies met all the eligibility criteria, where seven were included in the qualitative synthesis (narrative synthesis), and three were included in the meta-analysis.

**Figure 1:**
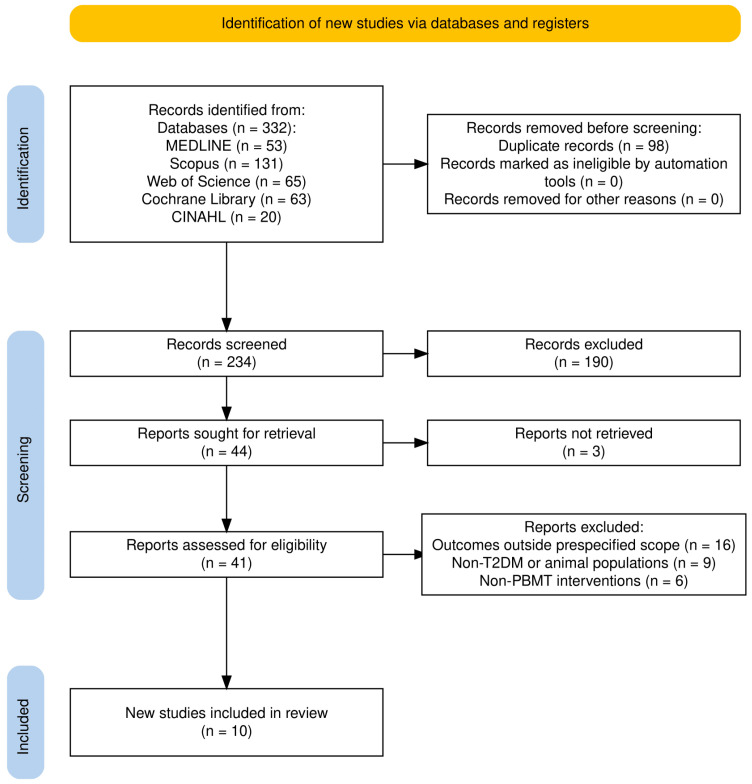
PRISMA 2020 flow diagram of the study selection process.

The study selection process is presented in the PRISMA flow diagram in Figure 1.

### 2.5. Data Extraction

Data from each included study were independently extracted by two reviewers (A.W. and C.D.M.S.) using a form created using Microsoft Office Excel 2010, with discrepancies resolved, when required, by a third reviewer (S.N.L.). Extracted information included the following: study identification and design (first author, year, title, and study design); participants characteristics (sample size, age (mean or range according to original trial report), sex distribution, duration of T2DM, and baseline glycated hemoglobin (HbA1c)); intervention parameters (PBMT type, wavelength, power, energy density, application site, session duration, number and frequency of sessions, and total treatment duration); comparator details (such as sham PBMT); and all outcome measures of interest, including metabolic, inflammatory, neurological, and other relevant outcomes, together with their reported time points. For each outcome, effect estimates, measures of variability, statistical significance, and their reporting format (mean/SD or median/IQR) were recorded, along with follow-up duration, adverse events reported, and relevant notes to aid interpretation. No automation tools were used for data extraction, and study authors were not contacted for additional data.

### 2.6. Risk of Bias Assessment

The Cochrane Risk of Bias version 2 (RoB2) tool was used to determine the quality of the included RCTs [16]. This tool assesses five domains, including: (1) bias arising from the randomization process; (2) bias due to deviations from intended interventions; (3) bias due to missing outcome data; (4) bias in measurement of the outcome; and (5) bias in selection of the reported result. Adaptations of the RoB 2 algorithm were applied for crossover and split-mouth designs according to the Cochrane guidance to account for the non-independence of repeated measurements within the same participant and the potential for period or carryover effects that could influence measurements.

Two reviewers (A.W. and C.D.M.S.) independently assessed the risk of bias for each study, with disagreements resolved through discussion by a third reviewer (S.N.L.). Overall, risk of bias judgements (“low risk”, “some concerns”, or “high risk”) were determined based on the RoB 2 decision rules, where the highest level of bias identified in any domain defined the overall rating.

No automation tools were used to perform risk of bias assessments, and the study authors were not contacted for clarification.

### 2.7. Data Synthesis and Statistical Analysis

All the extracted continuous outcome data were expressed as means ± standard deviations (SDs) in the same unit of measurement. Effect sizes were calculated as mean differences (MDs) with standard errors (SEs) and equivalent 95% confidence intervals (CIs). MDs were selected as the effect measures because all included studies for pooled analysis reported outcomes on the same unit and measurement scale. No minimal important difference or other interpretation thresholds were pre-specified, and no re-expression of effect measures was performed. When multiple time points were reported, the longest follow-up was selected for synthesis.

Meta-analyses were conducted for outcomes with data from at least two RCTs that reported comparable interventions, control groups, and sufficient statistical detail (mean, SD, and sample size). For eligible outcomes, a random-effects model (DerSimonian–Laird method) was applied to address clinical and methodological heterogeneity. CIs for pooled effects were calculated by using the Wald method, and between-study variance (τ^2^) was estimated using the DerSimonian–Laird approach. Statistical heterogeneity was determined by executing the Cochran’s Q test (χ^2^, *p*-value), the I^2^ statistic (low: 0–25%, moderate: 26–50%, substantial: 51–75%, considerable: >75%), and the τ^2^ estimate.

Where fewer than two studies were available for an outcome, or where reporting was too heterogeneous, results were summarized narratively. Studies excluded from pooling are identified in the Results Section, with reasons for exclusion, and their findings are described qualitatively (Section 3.4 and Section 3.5.2). No imputation of missing SDs or other effect size elements was performed, and no subgroup or sensitivity analyses were planned due to the small number of studies per outcome.

In addition, assessments of publication bias (e.g., funnel plots or statistical tests for small study effects) were not conducted. According to *The Cochrane Handbook for Systematic Reviews of Interventions* [14], subgroup analyses, sensitivity analyses, and publication bias assessments are not recommended when less than 10 studies are available for quantitative synthesis, as such analyses yield unstable and potentially misleading results. Consequently, heterogeneity was addressed with a priori restrictions on quantitative synthesis and qualitative comparison of study characteristics and PBMT parameters.

Statistical analyses were performed in R (version 4.4.1) by using the “metafor” package. All statistical tests were two-tailed, with significance set at *p* < 0.05.

#### Predefined Criteria for Quantitative Synthesis

To maintain biological and clinical interpretability, quantitative synthesis was restricted a priori to outcomes that met all of the following criteria: assessment in the same biological tissue or anatomical compartment, measurement of the same clinical endpoint by using comparable definitions, and reporting on identical or directly comparable measurement scales and units.

Based on these criteria, meta-analysis was limited to periodontal clinical outcomes, where PBMT was applied locally to periodontal tissues, outcomes were measured by using periodontal indices (CAL, PD, PI), and data were reported in consistent units (millimeters or validated indices).

Systemic metabolic, inflammatory, and neurological outcomes were not pooled because PBMT interventions varied substantially according to wavelength, energy density, duration, delivery modality, and application site, which resulted in heterogeneity that would preclude biologically meaningful or clinically interpretable pooled estimates.

This restriction reflects methodological necessity rather than outcome prioritization. The review objectives remained centered on the systemic, metabolic, inflammatory, and neurological effects of PBMT.

### 2.8. Certainty of Evidence

The certainty and quality of the evidence of the included studies were performed according to the Grading of Recommendations Assessment, Development, and Evaluation (GRADE) approach [17] in GRADEpro GDT [18].

For each outcome, the certainty of evidence was first rated as high for RCTs and downgraded by one or more levels when serious or very serious concerns were identified in any of the five domains: risk of bias, inconsistency, indirectness, imprecision, and publication bias. Downgrading for imprecision was applied when 95% confidence intervals included no effect or failed to meet optimal information size; inconsistency was considered when unexplained heterogeneity was substantial (I^2^ > 50%); indirectness was applied when populations, interventions, or outcomes differed from the review question; risk of bias followed the overall RoB 2 judgements; and publication bias when there was evidence or suspicion of selective reporting or small study effects.

The overall certainty rating was classified as high, moderate, low, or very low, to reflect the degree of confidence in the effect estimate. Two reviewers (A.W. and C.D.M.S.) independently conducted the assessments, and in cases of disagreement, a third reviewer was involved (S.N.L.).

## 3. Results and Discussion

### 3.1. Study Characteristics

A total of 10 RCTs met the eligibility criteria and were included in this review. The studies were conducted between 2015 and 2023 in six countries, including Brazil, Egypt, India, Kosovo, Saudi Arabia, and Turkey. All studies applied parallel-group or crossover randomized designs and assessed the effects of PBMT in adults diagnosed with T2DM.

Sample sizes ranged from 10 to 80 participants, with mean ages from 45 years to 63 years. Diabetes duration between participants varied from 1 year to 13 years. The proportion of female participants ranged from 0% to 80% and male participants from 20% to 100%.

The PBMT interventions differed in wavelength (630–1064 nm), power output (5–4000 mW), and energy density (0–288 J/cm^2^). Application sites included lower limb muscles (quadriceps, hamstrings, triceps surae), periodontal tissues, radial/wrist vessels, and oral mucosa. Session durations ranged from 15 s per site to approximately 90 min. Intervention frequency differed from single session applications to five consecutive daily sessions, or up to three sessions per week. Total intervention periods ranged from a single day to 12 months. Comparators included sham PBMT/LEDT devices, nonsurgical periodontal therapy (scaling and root planing) alone, or usual care with hypoglycemic medications.

Reported outcomes included metabolic measures (HbA1c, fasting glucose, random blood sugar (RBS), oral glucose tolerance (OGT), fasting C-peptide), inflammatory biomarkers (serum IL-6), and neurological outcomes (intraepidermal nerve fiber density, vibration perception threshold, neuropathy scores, and heart rate variability (HRV)). Some studies also reported periodontal indices (clinical attachment level (CAL), probing depth (PD), bleeding on probing (BOP), plaque index (PI), gingival index (GI), gingival bleeding index (GBI), IL-1β in gingival crevicular fluid (GCF), and salivary calprotectin).

The general characteristics of the included studies are summarized in Table 1, and details about the PBMT parameters with measured outcomes are presented in Table 2.

### 3.2. Risk of Bias Assessment

**Figure 2:**
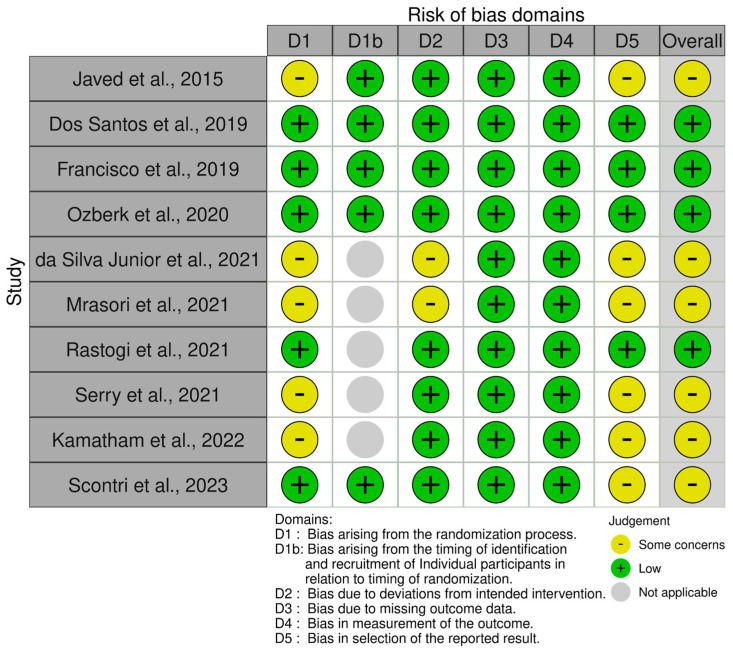
Summary of risk of bias assessment for the included RCTs [11,19,20,21,22,23,24,25,26,27].

We summarized the risk of bias assessment for the 10 included RCTs in Figure 2. Among them, four trials (40%) were judged as having “low risk of bias”, whereas six trials (60%) were assessed as having “some concerns”. No studies were rated as having a “high risk of bias”.

The most common domains that contributed to “some concerns” were Domain 1 (randomization process) and Domain 5 (selection of the reported result). Issues in Domain 1 were mainly associated with the incomplete report of allocation concealment and insufficient details about the blinding of participants, which may have influenced subjective outcomes (e.g., pain scores, self-reported neurological function). In Domain 5, the absence of a statistical analysis plan or trial registration and the incomplete report of the outcome measures raised concerns about potential selective reporting.

All studies were judged to be at “low risk” in Domain 2 (deviations from intended interventions) and Domain 4 (measurement of the outcome). In particular, most trials had minimal attrition and no evidence of protocol deviations that were likely to impact the outcomes. Here, measurement of the outcome was considered low risk for objective metabolic and inflammatory biomarkers (e.g., HbA1c, FPG, CRP, IL-6), but more prone to potential bias for subjective measures.

### 3.3. Primary Outcomes

In accordance with CONSORT principles, treatment effects are interpreted primarily on the basis of between-group comparisons. Within-group changes are reported descriptively only when between-group estimates were unavailable or not reported by the original trials. Metabolic, inflammatory, and neurological outcomes were predefined as the primary outcomes of this review. However, due to the limited number of eligible studies per outcome, substantial clinical and methodological heterogeneity (including differences in PBMT parameters, outcome definitions, and follow-up duration), and incomplete or incompatible statistical reporting, meta-analysis was not possible for these outcomes.

Therefore, results for all primary outcomes are summarized narratively based on the registered protocol and best practice guidance for evidence synthesis when pooling is not justified.

#### 3.3.1. Metabolic Outcomes

Six RCTs assessed at least one metabolic outcome, including HbA1c, FPG, OGT, RBS, and acute changes in blood glucose after intervention. Nevertheless, differences in outcome type, measurement period, intervention protocols, and statistical report precluded the inclusion for meta-analysis.

Metabolic outcomes are grouped into acute glycemic responses and longer-term glycemic control.

##### Acute Effects

Two crossover trials [11,21] investigated acute changes in blood glucose after a single PBMT or LEDT session. Both demonstrated significant short-term reductions in capillary or venous glucose relative to sham controls immediately after intervention. However, these effects were transient (≤12 h) and were not reflected in longer term glycemic indices such as HbA1c, which reflected acute physiological responses rather than sustained glycemic control.

##### Chronic Effects

One parallel-group RCT [26] examined the chronic effects of PBMT delivered via a laser watch over 12 weeks. Significant between-group reductions were observed in HbA1c (−1.18%), FPG (22.43 mg/dL), and OGT (−36.6 mg/dL) compared with standard care (all *p* < 0.0001). This was the only trial that reported chronic metabolic outcomes with sufficient statistical detail and between-group estimates suitable for effect size calculation. Therefore, pooling was not possible.

Other trials reported inconsistent findings. One trial [23] found no significant between-group differences in HbA1c or FPG following intravascular PBMT in addition to scaling and root planing, with results reported as medians (IQR), which limited compatibility with other trials. Kamatham et al. [27] reported significant within-group reductions in RBS at 8 weeks in both PBMT–SRP and SRP-only groups, but between-group differences were not statistically significant, and the use of random rather than fasting glucose further limited interpretability. Javed et al. [19] reported reductions in HbA1c relative to baseline (*p* < 0.05), but data were grouped across participants without a comparator group estimate, precluding assessment of treatment effect.

Overall, between-group effects on metabolic outcomes were inconsistent, and only one RCT reported sustained improvements suitable for quantitative interpretation.

#### 3.3.2. Inflammatory Outcomes

Three RCTs determined the effects of PBMT on inflammatory markers in individuals with T2DM and periodontitis. Nevertheless, the studies applied different inflammatory biomarkers and measurement methods, and reported data at different follow-up intervals, which prevented quantitative synthesis.

Mrasori et al. [24] assessed systemic inflammation by measuring serum interleukin-6 (IL-6) levels. After five consecutive daily PBMT sessions, a statistically significant between-group reduction in IL-6 was observed at 3 months compared with control (*p* = 0.001). Mean IL-6 levels declined from 11.54 ± 1.11 pg/mL to 11.02 ± 0.67 pg/mL in the intervention group.

Kamatham et al. [27], measured salivary calprotectin at baseline and 8 weeks after a single session of PBMT delivered in addition to SRP. Both the PBMT–SRP and SRP-only groups showed statistically significant within-group reductions. However, this reduction was greater in the PBMT group (*p* < 0.001).

In addition to systemic markers, one study [22] measured interleukin-1β (IL-1β) levels in GCF as a local inflammatory mediator in patients with T2D and periodontitis. PBMT was applied in addition to nonsurgical periodontal therapy and produced a significant reduction in IL-1β concentrations compared with the control groups at 3 months (*p* < 0.05), with the effect sustained at 6 months.

Overall, only one RCT reported a statistically significant between-group reduction in a systemic inflammatory biomarker, while other studies reported within-group or local inflammatory changes only.

#### 3.3.3. Neurological Outcomes

Two RCTs evaluated neurological outcomes, based on autonomic function, peripheral neuropathy symptoms, and nerve conduction parameters. Nevertheless, the studies were different in terms of intervention protocols, outcome measures, and follow-up periods, which did not enable quantitative synthesis.

Scontri et al. [11] investigated autonomic nervous system function by using HRV indices after a single PBMT session at different energy doses. Significant between-group improvements in several HRV parameters were observed compared with sham. However, effects were measured only up to 12 h post-intervention, and their clinical relevance beyond short-term autonomic changes is uncertain.

Rastogi et al. [25], assessed painful diabetic peripheral neuropathy using the visual analogue scale (VAS) for pain, the Norfolk Quality of Life–Diabetic Neuropathy questionnaire, and intraepidermal nerve fiber density (IENFD). Over 12 weeks, the PBMT resulted in a substantial between-group reduction in pain scores (−5.1 points) and improved quality of life (+15 points) compared with the sham. These changes exceeded established thresholds for clinical relevance. However, no significant between-group changes were observed in IENFD, which suggested that symptom improvement did not correspond to measurable nerve regeneration during the study period.

Overall, PBMT was associated with short-term autonomic modulation and clinically meaningful symptomatic relief in painful diabetic peripheral neuropathy, based on between-group comparisons. No between-group changes were observed in structural nerve measures such as IENFD.

### 3.4. Secondary Outcomes

Because periodontal outcomes were the only secondary outcomes to meet the predefined criteria for quantitative synthesis, including local application to the same tissue compartment, standardized clinical endpoints, and comparable measurement units, they are presented separately below. All prespecified primary outcomes are reported narratively in accordance with the registered protocol. The following subsections present the secondary outcomes, including pooled results for CAL, PD, and PI, and narrative summaries for BOP, GBI, and GCF.

#### 3.4.1. Periodontal Outcomes (Meta-Analysis)

We conducted three meta-analyses, where only periodontal clinical outcomes, including CAL, PD, and PI, were suitable for quantitative synthesis, as they were assessed in at least two studies, with comparable units and scales. Random-effects models were applied in all cases.

##### Clinical Attachment Level

Four RCTs [20,22,23,27] reported the effect of PBMT on CAL. The study conducted by Da Silva Júnior et al. [23] could not be included in the pooled analysis because their results were reported as medians with interquartile ranges rather than means with standard deviations.

The remaining three RCTs [20,22,27] measured CAL in millimeters using comparable methodology, with follow-up periods that ranged from 8 weeks to 12 months.

**Figure 3:**
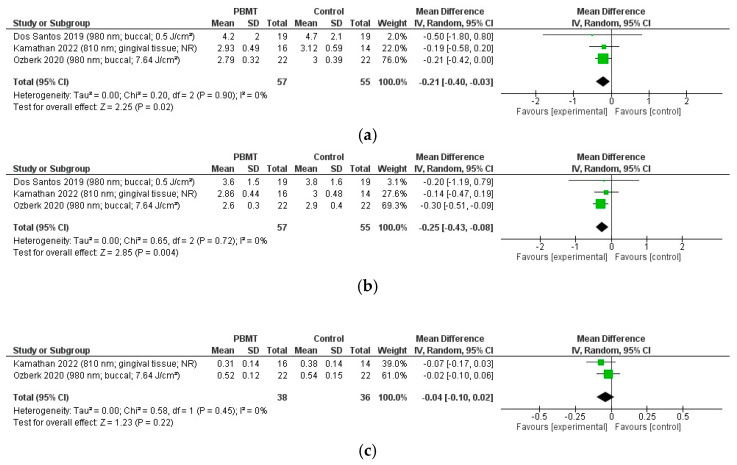
Forest plots of pooled effects for periodontal outcomes: (**a**) CAL, (**b**) PD, and (**c**) PI between the intervention and control groups [20,22,27]. Bold formatting indicates the pooled overall effect estimate. Abbreviations: CI, confidence interval; df, degrees of freedom; IV, inverse variance; SD, standard deviation; NR, not reported; Tau^2^, between study variance; Chi^2^, Cochran’s Q statistic; Z, overall effect test statistic.

The pooled analysis showed a significant improvement in CAL with PBMT when compared with control interventions (mean difference = −0.21 mm, 95% CI: −0.39, −0.03; *p* < 0.05) (Figure 3a). Individual trial effect estimates ranged from −0.19 mm to −0.50 mm. Heterogeneity between the articles was low (I^2^ = 0%, *p* = 0.90).

The certainty of evidence for this outcome was low according to GRADE (Table 3), because the evidence was downgraded for risk of bias (two studies low risk, one with some concerns) and imprecision (small total sample size, 95% CI narrowly excluding no effect).

##### Probing Depth

Five RCTs [19,20,22,23,27] reported the effect of PBMT on PD. Two trials could not be included in the meta-analysis: Javed et al. [19] presented PD as the percentage of sites with PPD ≥ 4 mm rather than mean PD; Da Silva Júnior et al. [23] reported medians.

The remaining three RCTs [20,22,27] measured PD in millimeters by using a comparable methodology, with follow-up periods ranging from 8 weeks to 12 months.

The pooled analysis demonstrated a significant reduction in PD with PBMT when compared with control interventions (mean difference = −0.25 mm, 95% CI: −0.43, −0.07; *p* < 0.05) (Figure 3b). Individual trial effect estimates ranged from −0.14 mm to −0.30 mm. Statistical heterogeneity was low (I^2^ = 0%; τ^2^ = 0; Q = 0.65, *p* = 0.72).

According to GRADE, the certainty of the evidence for this outcome was low (Table 3) as it was downgraded for risk of bias (two studies low risk, one with some concerns), and imprecision (small total sample size; 95% CI not far from the null).

##### Plaque Index

Four RCTs [19,20,22,27] reported the effect of PBMT on PI. However, two trials [19,20] were excluded from pooling: Javed et al. [19] presented PI as the percentage of sites with plaque and reported only means with ranges (no SD), whereas Dos Santos et al. [20] reported PI as the percentage of sites with supragingival biofilm with no mean or SD provided.

The remaining two RCTs [22,27] used comparable measurement scales (Silness–Löe index), with follow-up periods ranging from 8 weeks to 6 months.

The pooled analysis found no significant difference in PI between PBMT and control interventions (mean difference = −0.04, 95% CI: −0.10, 0.02; *p* > 0.05) (Figure 3c). Individual trial effect estimates ranged from −0.02 to −0.07. Statistical heterogeneity was low (I^2^ = 0%; τ^2^ = 0; Q = 0.58, *p* = 0.45).

According to GRADE, the certainty of the evidence for this outcome was low (Table 3), because it was downgraded for risk of bias (one study low risk, one with some concerns) and for imprecision (small sample size and 95% CI including no effect).

#### 3.4.2. Periodontal Outcomes (Narrative)

Several trials reported periodontal outcomes that could not be included in the meta-analysis due to incompatible outcome definitions, different measurement scales, lack of summary statistics, or insufficient reported data.

##### Bleeding on Probing and Gingival Bleeding Index

Four RCTs assessed the bleeding-related periodontal outcomes. Da Silva Júnior et al. [23] measured GBI and BOP at baseline and 4 months after intravascular PBMT and SRP, where both groups improved significantly (*p* < 0.05), but between-group differences were not significant. Özberk et al. [22] measured BOP at 1, 3, and 6 months in a split-mouth design, where treated sites showed significant within-group reductions over time, but between-group comparisons and complete summary statistics were not reported. Dos Santos et al. [20] expressed BOP as the percentage of bleeding sites, where both PBMT and control pockets improved over 3 to 12 months, with no significant between-group differences. Kamatham et al. [27] reported significant BOP and GBI reductions at 8 weeks in PBMT and SRP compared with SRP alone (*p* < 0.05). All of these studies suggest PBMT can reduce gingival bleeding indices when used in addition to SRP; however, differences in scoring systems, follow-up times, and incomplete data limited the certainty of the evidence.

##### Gingival Crevicular Fluid

Özberk et al. [22] also assessed GCF volume at baseline and multiple follow-ups to 6 months. PBMT-treated sites had significantly lower GCF volumes than controls at all post-treatment timepoints (*p* < 0.05), with reductions evident from 1 week and maintained over 6 months. Within-group decreases were seen in both groups, but the effect was greater with PBMT.

### 3.5. Discussion

This systematic review and meta-analysis evaluated the effects of PBMT on metabolic, inflammatory, neurological outcomes in adults with T2DM. These were the prespecified primary outcomes of the review. Due to heterogeneity in intervention protocols, outcome definitions, and reporting quality, these primary outcomes could not be pooled quantitatively and were therefore synthesized narratively.

Although periodontal outcomes were secondary and not predefined as primary endpoints, they were consistently reported with sufficient methodological comparability in multiple trials. In particular, they were applied locally to the same tissue compartment (periodontal tissues); the outcomes reflected the same clinical constructs, and measurements were reported in comparable units. Therefore, they represented the only outcome category suitable for meta-analysis. Quantitative synthesis of these secondary outcomes was conducted to avoid discarding homogeneous data, while maintaining transparency about outcome hierarchy.

#### 3.5.1. Metabolic Outcomes

Metabolic outcomes were investigated in six RCTs, but differences in outcome measures, intervention protocols, and reporting formats precluded meta-analysis. In general, the evidence was inconsistent. Two crossover studies [11,21] reported only acute reductions in glucose after single PBMT or LEDT sessions. Although biologically plausible via enhanced mitochondrial respiration, increased ATP production, and glucose uptake via GLUT4 translocation [28,29], most evidence from these mechanisms comes from animal models, and long-term improvements in glycemic control in humans is uncertain.

In contrast, Serry et al. [26] reported an improvement in HbA1c and glucose outcomes after 12 weeks of PBMT delivered via a laser watch. These changes exceeded the ≥0.5% HbA1c decrease value considered to be clinically significant [30], thus suggesting a potential effect. However, this trial is the only RCT to demonstrate these effects, and replication is lacking.

Other studies reported mixed results. They showed either modest within-group changes in RBS [27] or no significant between-group differences in HbA1c or fasting glucose [23]. Finally, Javed et al. [19] reported HbA1c reductions, but the absence of a comparator group estimates limited interpretations about treatment efficacy.

In summary, these findings demonstrate the uncertainty about PBMT’s role in metabolic control. The differences in PBMT parameters (wavelength, energy density, application site), study design, and follow-up likely contributed to the variability. Mechanistically, PBMT may plausibly enhance mitochondrial function, reduce oxidative stress, and improve insulin sensitivity via GLUT4 translocation, but most of this evidence is derived from preclinical models. Current clinical data are insufficient to confirm sustainable metabolic benefits in T2DM; thus, larger and longer term RCTs with uniform PBMT approaches are required.

##### Mechanistic Considerations for Systemic Metabolic Effects of Local PBMT

PBMT exerts its biological effects at the site of application through photoreception by mitochondrial chromophores, such as cytochrome c oxidase, which results in increased ATP production, modulation of reactive oxygen species, and reduction in local inflammatory signaling [9,31]. In periodontal tissues, repeated PBMT application may decrease local inflammation and oxidative stress, which could theoretically influence systemic metabolism through reduced cytokine spillover, improved endothelial function, or modulation of insulin sensitivity [32,33,34,35]. PBMT applied to skeletal muscle has been hypothesized to promote mitochondrial efficiency and glucose uptake via insulin-dependent pathways such as GLUT4 translocation [28].

In both models, the proposed mechanism requires that local biological effects are transmitted via systemic mediators to influence whole body glucose homeostasis. However, HbA1c and other metabolic indices reflect whole-body glycemic exposure over weeks–months. In contrast, localized, low-dose, short-duration PBMT protocols, as applied in the current trials, are likely to affect an insufficient tissue volume and exposure duration to elicit sustained systemic metabolic effects.

#### 3.5.2. Inflammatory Outcomes

Only one RCT investigated systemic inflammatory biomarkers in individuals with T2DM. Mrasori et al. [24] reported a significant reduction in serum IL-6 levels three months after five daily PBMT sessions compared with the sham, which suggests a potential systemic anti-inflammatory effect. IL-6 is a relevant biomarker in T2DM, as high levels contribute to insulin resistance, endothelial dysfunction, and vascular complications [36].

This result is in agreement with those of preclinical studies, which showed that PBMT downregulates nuclear factor kappa B (NF-κB) activation, which leads to reduced expression of pro-inflammatory cytokines such as IL-6 and tumor necrosis factor-alpha (TNF-α), while modulating oxidative stress and improving insulin sensitivity [20,37].

The gap between strong mechanistic evidence and limited clinical confirmation likely reflects the methodological flaws of current trials, including small sample sizes, short follow-up, and limited biomarker panels. Differences in disease stage and comorbidities may further influence patient responsiveness to PBMT, which further contributes to the inconsistent outcomes in studies.

In summary, early evidence suggests that PBMT could reduce systemic inflammation in T2DM, but the certainty of evidence is very low. Therefore, larger RCTs that include broader biomarker panels (e.g., IL-6, TNF-α, CRP, and markers of oxidative stress), with longer observation periods are required to determine whether PBMT can reduce systemic inflammation and thus improve downstream clinical outcomes in T2DM.

#### 3.5.3. Neurological Outcomes

Neurological outcomes were evaluated in two RCTs, but differences in intervention protocols, outcome measures, and follow-up durations precluded meta-analysis. In general, PBMT appears to provide short-term symptomatic and autonomic benefits, but evidence for nerve repair is lacking.

Scontri et al. [11] reported acute improvements in HRV indices after a single PBMT session, which suggested short-term alteration of the autonomic nervous system. However, these effects only lasted up to 12 h and their clinical significance is uncertain. Similar short-term autonomic changes have been reported in non-diabetic populations with musculoskeletal pain [38], which demonstrates that PBMT may impose general neuromodulatory rather than disease effects.

In contrast, Rastogi et al. [25] found substantial improvements in painful diabetic peripheral neuropathy, where PBMT reduced pain by 5.1 points on the VAS, and improved quality of life scores by 15 points after 12 weeks compared with sham. These changes exceeded the 1.7–2-point threshold on the VAS considered clinically meaningful [39], which indicate significant symptomatic relief. Nevertheless, no changes were observed in IENFD, which suggests that improvements were not caused by nerve regeneration during the study timeframe.

These results align with those of previous research, which reported that PBMT may provide short-term relief in neuropathic symptoms; however, these effects depend on treatment parameters (e.g., wavelength, dosage, and area of application) and are not sustained long-term [12,40,41]. PBMT can improve neuropathic symptoms by neuromodulation rather than immediate anatomical repair, including downregulation of inflammatory cytokines, promotion of growth factor expression, and modulation of intracellular pathways such as MAPK [12,42]. These mechanisms explain the symptomatic improvements observed in Rastogi et al. [25], regardless of unchanged IENFD, and the acute autonomic modulation reported by Scontri et al. [11].

In summary, the current evidence suggests that PBMT may provide short-term symptomatic and autonomic improvements in T2DM neuropathy, but long-term effects on nerve regeneration or conduction have not yet been demonstrated. Current trials are small, heterogeneous, and limited by short follow-up durations. Therefore, further RCTs that include established functional and morphological neurological outcomes, and longer intervention periods are required to determine whether PBMT can provide long-term neuroprotective benefits in diabetic neuropathy.

#### 3.5.4. PBMT Parameters and Outcome Variability

Although formal stratified or subgroup analyses were not performed, qualitative comparison of PBMT parameters across studies was conducted to contextualize heterogeneity. Acute metabolic or autonomic effects were reported predominantly in studies that applied single or short PBMT sessions to peripheral tissues [11,21,27], while periodontal benefits were observed following repeated local application over several weeks [20,22,23,27]. Studies that used near infrared wavelengths and repeated exposure tended to report more local tissue effects [11,22,25], while systemic outcomes remained inconsistent regardless of wavelength or dose [11,19,21,23,26]. Although formal stratified analyses were not feasible, this comparison suggests that PBMT efficacy in T2DM may be dependent on application site, treatment duration, and parameter selection.

#### 3.5.5. Periodontal Outcomes

##### CAL, PD, and PI (Meta-Analysis)

Periodontal outcomes were the only category with sufficient homogeneity for meta-analysis. Pooled results from three RCTs showed statistically significant improvements in CAL (0.21 mm) and PD (0.25 mm) with PBMT compared with control interventions, both with low heterogeneity (I^2^ = 0%). No significant effect of PBMT was observed for PI. The certainty of evidence was graded as low due to the small sample sizes and risk of bias.

These findings are consistent with a previous systematic review of 17 RCTs [43], which concluded that PBMT used as an adjunct to NSPT significantly promoted periodontal healing compared with NSPT alone. However, heterogeneity and methodological concerns limited the certainty of evidence. Evidence from RCTs in T2DM populations further supports this. Dos Santos et al. [20] reported that adjunctive PBMT reduced the proportion of moderate PD pockets and lowered the frequency of sites with PD of 5–6 mm compared with SRP alone. Furthermore, an umbrella review and meta-analysis by Al Malak et al. [44], which involved 19 RCTs of diode laser therapy adjunctive to SRP in chronic periodontitis, reported modest PD reductions (0.30 mm at three months; 0.32 mm at six months) and CAL gains (0.35 mm at three months). These pooled estimates closely mirror our findings, suggesting that PBMT’s benefits are small but induce consistent periodontal improvements in populations with and without T2DM.

Although statistically significant, the pooled reductions of 0.2 to 0.3 mm in CAL and PD are unlikely to be clinically relevant in isolation, since improvements of ≥1 mm are generally considered pertinent for long-term periodontal stability [45]. Nonetheless, they may be relevant for populations with impaired healing capacity, such as individuals with T2DM, where even small improvements in periodontal health may contribute to reduced systemic inflammatory burden [46] and better disease management [47].

In contrast, PBMT showed no effect on PI (*p* > 0.05), which mirrors prior evidence that shows its main benefits are related to host tissue healing and modulation of the inflammatory response rather than mechanical removal of supragingival plaque [48]. This suggests that reductions in PI depend predominantly on scaling and patient oral hygiene practices.

In summary, PBMT was associated with statistically significant but small improvements in CAL and PD. However, because of the low certainty of the evidence, small effect sizes, and methodological limitations in the contributing trials, these findings should not be interpreted as reliable or clinically definitive and are unlikely to be clinically meaningful in isolation.

##### Periodontal Inflammatory Measures (Narrative Synthesis)

In four RCTs, PBMT used adjunctively with SRP reduced gingival bleeding indices (BOP, GBI), GCF volume, and local inflammatory mediators (salivary calprotectin, GCF IL-1β). However, between-group differences were inconsistent, and reporting was often incomplete, which precluded meta-analysis.

Da Silva Júnior et al. [23] found improvements in BOP and GBI at four months in both PBMT and control groups, but no significant between-group differences. In contrast, Kamatham et al. [27] reported significantly greater reductions in BOP, GBI, and salivary calprotectin levels at eight weeks in the PBMT group compared with SRP alone. Özberk et al. [22] showed that PBMT significantly reduced GCF IL-1β concentrations up to six months after treatment; however, summary statistics were not available for BOP. Similarly, observed reductions in bleeding sites over 3 to 12 months, with no significant differences found between PBMT and the control groups.

In summary, these findings suggest that PBMT may promote resolution of gingival inflammation when used as an adjunct to SRP. Nevertheless, the overall certainty of evidence is limited by short follow-up periods, heterogeneous scoring systems, small sample sizes, and incomplete reporting. Therefore, uniform definitions and reporting of periodontal inflammatory indices are needed to establish whether PBMT provides adjunctive benefits.

#### 3.5.6. Strengths and Limitations

This is the first systematic review and meta-analysis to assess evidence on the use of PBMT for metabolic, inflammatory, neurological, and periodontal outcomes in adults with T2DM, which is an underexplored but important therapeutic domain. The review followed a registered protocol (PROSPERO CRD420251083550), adhered to PRISMA 2020 guidelines, and applied a literature search strategy with no date restrictions. Study selection, and risk of bias assessments were performed in duplicate to reduce subjectivity, while meta-analysis and GRADE assessments were applied where feasible to promote reliability. The deliberate restriction of meta-analysis to biologically and clinically comparable periodontal outcomes avoided inappropriate pooling between heterogeneous PBMT modalities and reduced the risk of producing misleading pooled estimates.

In addition, the limited number of studies eligible for quantitative synthesis precluded formal subgroup analyses, sensitivity analyses, and assessments of publication bias. Although this restricts the ability to explore effect modifiers statistically, it reflects the current state of the evidence base rather than a limitation of the review methodology.

Safety outcomes were inconsistently reported between the included trials. Most studies reported no adverse events associated with PBMT, while others did not report safety outcomes explicitly. No serious adverse events were described. However, the absence of systematic safety reporting limits conclusions about tolerability and long-term safety in T2DM populations.

Nevertheless, some limitations of the review process should be acknowledged. First, only English language publications were included, which may have introduced language bias and excluded relevant non-English evidence. Second, study authors were not contacted for missing or incomplete data, which may have limited quantitative synthesis. This decision reflects the pragmatic constraints of the review, and it is acknowledged that author contact could have expanded pooled analyses. Finally, prospective trial registries were not searched beyond major databases, which may increase the risk of publication bias.

In addition, limitations of the evidence base itself reduce confidence in the findings. Only 10 RCTs were included, most with small sample sizes, short follow-up, and heterogeneous PBMT approaches, which limited pooled analyses and dose and response assessment. Reporting was often incomplete, with selective outcome reporting and insufficient statistical detail. Risk of bias was judged “low” in only 40% of trials, with the remainder rated as “some concerns”. Together, these factors contributed to the low certainty of evidence between outcomes.

#### 3.5.7. Clinical and Research Implications

From a clinical perspective, the current evidence suggests that PBMT may be associated with small, statistically significant adjunctive periodontal effects when used alongside scaling and root planing in individuals with T2DM. However, the certainty of evidence is low, and the magnitude of benefit in CAL and PD were small (<0.3 mm) and unlikely to be clinically meaningful when used in isolation. Therefore, PBMT should not be considered an evidence-based therapeutic adjunct at present, but an experimental or supportive intervention pending confirmation in larger, well-designed trials.

For metabolic, inflammatory, and neurological outcomes, the evidence base is too limited and heterogeneous to support translation into practice. A single trial reported significant reductions in HbA1c after 12 weeks of PBMT, but replication is absent. Likewise, early findings suggest PBMT may reduce systemic inflammatory biomarkers and provide short-term relief of neuropathic pain, but these observations are not supported by larger RCT data, as has been explored in patients with fibromyalgia [49,50,51,52]. Therefore, clinicians should view these effects as preliminary and promising, but not sufficient for practice.

Future research should focus on overcoming the methodological limitations of current trials. Larger, multicenter RCTs with longer follow-up periods and established PBMT approaches (e.g., wavelength, dosage frequency, and application sites) are essential to determine whether benefits observed in pilot studies can be reproduced. Uniform outcome measures for systemic biomarkers and neurological endpoints will facilitate pooling of data in future meta-analyses and help establish clinical relevance. In addition, mechanistic studies in human populations are needed to confirm whether the pathways demonstrated in preclinical models (e.g., mitochondrial activation, GLUT4 translocation, NF-κB inhibition, neurotrophic factor upregulation) translate into long-term physiological and clinical effects.

## 4. Conclusions

In conclusion, this systematic review and meta-analysis found that PBMT is associated with statistically significant but small improvements in selected periodontal outcomes, based on low-certainty evidence. These effects are unlikely to be clinically meaningful in isolation. The evidence for metabolic, inflammatory, and neurological effects is limited, inconsistent, and preliminary. Further large-scale, well-designed RCTs are required before PBMT can be recommended for clinical use in diabetes care.

## Figures and Tables

**Table 1:** General characteristics of RCTs included in the review.

Study	Country	Design Type	Sample Size (I/C)	Mean Age (Years)	Average Diabetes Duration (Years)	Gender (M/F %)
Javed et al., 2015 [19]	Saudi Arabia	Split-mouth pilot RCT (single-masked)	44 (22/22)	54	7	86/14
Dos Santos et al., 2019 [20]	Brazil	Split-mouth RCT (double-blind)	19	52	≥5	13/36
Francisco et al., 2019 [21]	Brazil	Crossover RCT (double-blind)	16 (crossover)	55	9	100/0
Özberk et al., 2020 [22]	Turkey	Split-mouth RCT (single-blind)	22 (split-mouth)	45	6	46/55
da Silva Júnior et al., 2021 [23]	Brazil	Parallel RCT (blinded)	21 (10/11)	45–77 (range)	Not reported	24/76
Mrasori et al., 2021 [24]	Kosovo	Parallel RCT (2-arm)	80 (40/40)	35–60 (range)	Not reported	Not reported
Rastogi et al., 2021 [25]	India	Parallel RCT (sham-controlled)	30 (15/15)	59	12	36/64
Serry et al., 2021 [26]	Egypt	Parallel RCT	60 (30/30)	54	>1	46/14
Kamatham et al., 2022 [27]	India	Parallel RCT (4-arm design)	60 (with subgroups: 14–16 in DM arms)	46	Not reported	57/43
Scontri et al., 2023 [11]	Brazil	Crossover RCT	10 (crossover)	63	6	30/70

Abbreviations, C: Control group; DM: Diabetes mellitus; F: Female; I: Intervention group; M: Male; RCT: Randomized controlled trial. Age is reported as mean years where available; ranges are shown when studies reported age ranges only. Diabetes duration is presented as reported in the original trials. “Not reported” indicates unavailable data.

**Table 2:** PBMT parameters, comparators, and outcome measures of included RCTs.

Study	PBMT Parameters (Reported/Not Reported): Wavelength [nm], Power [mW], Irradiance [mW/cm^2^], Energy Density [J/cm^2^], Spot Size [cm^2^], Application Site, Duration, Sessions/Week, Total Duration	Comparator	Outcome Type	Outcome Measures	Follow-Up
Javed et al., 2015 [19]	Nd:YAG laser: 1064 nm; 4000 mW (4 W); 1.43 × 10^6^ mW/cm^2^ (fiber tip); energy density NR, spot size: NR; PPD ≥ 4 mm; 60–120 s per tooth; single session; single application	SRP alone (split-mouth control sites)	Metabolic; Periodontal	HbA1c (baseline and follow-up); PI; BOP; PD; CAL;	3 months
Dos Santos et al., 2019 [20]	Diode laser: 660 nm; 30 mW; 1.1 W/cm^2^ (1100 mW/cm^2^); 22 J/cm^2^; 0.028 cm^2^; buccal and lingual sites of PPD ≥ 5 mm; 20 s; single session; single application	SRP alone (split-mouth control)	Periodontal	PD; CAL; BOP; PI; Proportion of sites by PD category;	12 months
Francisco et al., 2019 [21]	LEDT: 850 nm; 75 mW per diode; 375 mW/cm^2^; 15 J/cm^2^ per diode (150 J total); 0.2 cm^2^; quadriceps and triceps surae (bilateral skin contact); 40 s per site; single session; single application	Placebo (LEDT device off)	Metabolic; Cardiopulmonary	Fasting glucose and lactate levels (before and after exercise), HbA1c, VO_2_ dynamics, heart rate, cardiac output, and muscle deoxyhemoglobin	Acute (post-exercise)
Özberk et al., 2020 [22]	GaAlAs diode laser: 980 nm; 400 mW; irradiance NR; 7.64 J/cm^2^ per site; 0.785 cm^2^; buccal surfaces of periodontal tissues (maxilla and mandible); 15 s per tooth; 4 sessions (day 0, 1, 3, 7); short-term adjunct to NSPT	SRP alone (contralateral quadrant)	Periodontal; Biochemical	PD; CAL; GI; PI; GCF volume; IL-1β (GCF)	6 months
Da Silva Júnior et al., 2021 [23]	Modified intravascular laser irradiation of blood: 660 nm; 100 mW; irradiance NR; 6.4 J/cm^2^; spot size NR; radial artery (wrist, ILIB-M via silicone bracelet, skin contact); 15–30 min; 10 sessions (every other day for 2 weeks)	SRP only (conventional periodontal therapy)	Metabolic; Periodontal	HbA1c; FPG (baseline and follow-up); VPI; GBI; BOP; PD; CAL	4 months
Mrasori et al., 2021 [24]	Diode laser: 660 nm; 10 mW; irradiance NR; energy density NR; spot size NR; gingival application (5 quadrants); 8 min per day; 5 sessions (consecutive days)	Periodontal therapy only	Inflammatory	Serum IL-6	3 months
Rastogi et al., 2021 [25]	MIRE therapy: 890 nm; power NR; irradiance NR; 1.95 J/cm^2^/min (58.5 J/cm^2^ per 30 min session); spot size NR; distal posterior and anterior leg, plantar arch; 30 min; 36 sessions (3/week for 12 weeks)	Sham MIRE (device off)	Neurological; QoL	VAS pain score; IENFD; MNSI; DNS; NDS; VPT; Norfolk QoL-DN	12 weeks
Serry et al., 2021 [26]	LLLT: 650 nm; 5 mW per beam; 160 mW/cm^2^; 288 J/cm^2^; 0.03 cm^2^; radial and ulnar vessels (wrist); 30 min; 36 sessions (3/week for 12 weeks)	Usual care (metformin and sulphonylurea only)	Metabolic	HbA1c; FPG; OGTT; Fasting C-peptide	12 weeks
Kamatham et al., 2022 [27]	Diode laser: 630 = 670 nm; 4 W; irradiance NR; energy density NR; spot size NR; gingival margin to pocket base and lateral/apical decontamination; duration NR; single session; single application	SRP alone (and SRP with LLLT)	Metabolic; Inflammatory; Periodontal	RBS (baseline and follow-up); Salivary calprotectin; PI; BOP; PPD; CAL	8 weeks
Scontri et al., 2023 [11]	Infrared LED: 830 ± 20 nm; 80 mW per array; 114.28 mW/cm^2^; 5.71 J/cm^2^ (100 J) or 13.71 J/cm^2^ (240 J); 0.2 cm^2^ per LED; skeletal muscles (quadriceps femoris, hamstrings, triceps surae, ventral upper arm & forearm; bilateral, contact mode); 50 s (100 J) or 120 s (240 J) per site; single session;	Sham PBMT (device off) and hypoglycemic medication	Metabolic and Neurological	Capillary glycemia; HRV indices	12 h after PBMT

BOP, bleeding on probing; CAL, clinical attachment level; DNS, Diabetic Neuropathy Symptom score; FPG, fasting plasma glucose; GBI, gingival bleeding index; GCF, gingival crevicular fluid; GI, gingival index; HbA1c, glycated hemoglobin; HRV, heart rate variability; IENFD, intraepidermal nerve fiber density; IL-1β, interleukin-1 beta; IL-6, interleukin-6; ILIB-M, modified intravascular laser irradiation of blood; J/cm^2^, joules per square centimeter; LEDT, light emitting diode therapy; LLLT, low level laser therapy; MIRE, monochromatic infrared energy; MNSI, Michigan Neuropathy Screening Instrument; NDS, Neuropathy Disability Score; NSPT, Non-Surgical Periodontal Therapy; NR, not reported; Nd:YAG, neodymium-doped yttrium aluminum garnet; nm, nanometer; OGTT, oral glucose tolerance test; PBMT, photobiomodulation therapy; PD, probing depth; PI, plaque index; PPD, probing pocket depth; QoL, quality of life; QoL-DN, Norfolk Quality of Life = Diabetic Neuropathy questionnaire; RBS, random blood sugar; SRP, scaling and root planing; VAS, visual analog scale; VO_2_, oxygen uptake; VPI, visible plaque index; VPT, vibration perception threshold; W, watt. Baseline metabolic indices (e.g., HbA1c, fasting plasma glucose) were reported only in studies that included metabolic or systemic outcomes. Periodontal only trials did not consistently assess baseline metabolic parameters.

**Table 3:** Summary of findings (GRADE certainty of evidence) for periodontal outcomes.

Outcomes	Mean Difference (95% CI)	Number of Participants (Studies)	Certainty of Evidence (GRADE)
CAL	−0.21 (−0.39 to −0.03)	112 (3 RCTs)	⊕⊕◯◯ Low ^a,b^
PD	−0.25 (−0.43, −0.07)	112 (3 RCTs)	⊕⊕◯◯ Low ^a,b^
PI	−0.04 (−0.10, 0.02)	74 (2 RCTs)	⊕⊕◯◯ Low ^a,b^

Abbreviations: CAL, clinical attachment level; CI, confidence interval; GRADE, Grading of Recommendations Assessment, Development, and Evaluation; PD, probing depth; PI, plaque index; RCTs, randomized controlled trials. Explanations: ^a^ downgraded one level for risk of bias due to blinding and reporting concerns in some studies; ^b^ downgraded one level for imprecision owing to small total sample size and wide 95% CI including or approaching no effect. Certainty of evidence was assessed by using the GRADE approach, where ⊕⊕⊕⊕ indicates high certainty, ⊕⊕⊕◯ moderate certainty, ⊕⊕◯◯ low certainty, and ⊕◯◯◯ very low certainty.

## Data Availability

No new data were created or analyzed in this study. Data sharing is not applicable to this article.

## Abbreviations

The following abbreviations are used in this manuscript:

ATP	adenosine triphosphate
BOP	bleeding on probing
CAL	clinical attachment level
CI	confidence interval
CIs	confidence intervals
CINAHL	Cumulative Index to Nursing and Allied Health Literature
CRP	C-reactive protein
DM	diabetes mellitus
DNS	Diabetic Neuropathy Symptom score
FPG	fasting plasma glucose
GBI	gingival bleeding index
GCF	gingival crevicular fluid
GI	gingival index
GRADE	Grading of Recommendations Assessment, Development and Evaluation
HbA1c	glycated hemoglobin
HRV	heart rate variability
IENFD	intraepidermal nerve fiber density
IL-1β	interleukin-1 beta
IL-6	interleukin-6
ILIB-M	modified intravascular laser irradiation of blood
IQR	Interquartile range
J/cm^2^	joule per square centimeter
LED	light-emitting diode
LEDT	light-emitting diode therapy
LLLT	low-level laser therapy
MD	mean difference
MIRE	monochromatic infrared energy
MNSI	Michigan Neuropathy Screening Instrument
NDS	Neuropathy Disability Score
Nd:YAG	neodymium-doped yttrium aluminum garnet
NF-κB	nuclear factor kappa B
nm	nanometer
NSPT	nonsurgical periodontal therapy
OGTT	oral glucose tolerance test
PBMT	photobiomodulation therapy
PD	probing depth
PI	plaque index
PICOS	Population, Intervention, Comparison, Outcomes, and Study design
PPD	probing pocket depth
PRISMA	Preferred Reporting Items for Systematic Reviews and Meta-Analyses
PROSPERO	International Prospective Register of Systematic Reviews
QoL	quality of life
QoL-DN	Norfolk Quality of Life–Diabetic Neuropathy questionnaire
RBS	random blood sugar
RCT	randomized controlled trial
RCTs	randomized controlled trials
RoB2	Cochrane Risk of Bias tool version 2
SD	standard deviation
SE	standard error
SRP	scaling and root planing
T2DM	Type 2 diabetes mellitus
TNF-α	tumor necrosis factor alpha
VAS	visual analog scale
VO_2_	oxygen uptake
VPI	visible plaque index
VPT	vibration perception threshold
W	Watt
τ^2^	between-study variance

## Appendix A

**Table A1:** Details of the search strategy.

Database	Search Strategy
MEDLINE	(“Diabetes Mellitus, Type 2” [Mesh] OR “type 2 diabetes” OR T2DM OR “adult onset diabetes” OR “non-insulin dependent diabetes”) AND (“Photobiomodulation” OR “photobiomodulation therapy” OR PBMT OR “low level laser therapy” OR LLLT OR “light-emitting diode therapy” OR LEDT OR “red light therapy” OR “cold laser”) AND ((“glycated hemoglobin” OR HbA1c OR “fasting glucose” OR insulin OR HOMA-IR OR OGTT OR “oral glucose tolerance” OR “glycemic control”) OR (“inflammatory markers” OR CRP OR cytokine* OR “tumor necrosis factor” OR TNF-alpha OR interleukin* OR IL-6 OR IL-1beta OR oxidative stress OR ROS) OR (“neuropathy” OR “nerve conduction” OR “neuropathy symptom score” OR “autonomic dysfunction” OR “heart rate variability” OR neuroinflammation OR BDNF OR NGF OR “quality of life”))
Scopus	TITLE-ABS-KEY((“type 2 diabetes” OR T2DM OR “adult onset diabetes” OR “non-insulin dependent diabetes”)) AND TITLE-ABS-KEY((“photobiomodulation” OR “photobiomodulation therapy” OR PBMT OR “low level laser therapy” OR LLLT OR “light emitting diode therapy” OR LEDT OR “red light therapy” OR “cold laser”)) AND TITLE-ABS-KEY((“glycated hemoglobin” OR HbA1c OR “fasting glucose” OR insulin OR HOMA-IR OR OGTT OR “oral glucose tolerance” OR “glycemic control” OR “inflammatory markers” OR CRP OR cytokine* OR “tumor necrosis factor” OR TNF-alpha OR interleukin* OR IL-6 OR IL-1beta OR oxidative stress OR ROS OR neuropathy OR “nerve conduction” OR “neuropathy symptom score” OR “autonomic dysfunction” OR “heart rate variability” OR neuroinflammation OR BDNF OR NGF OR “quality of life”)))
Web of Science	TS = (“type 2 diabetes” OR T2DM OR “adult onset diabetes” OR “non-insulin dependent diabetes”) AND TS = (“photobiomodulation” OR “photobiomodulation therapy” OR PBMT OR “low level laser therapy” OR LLLT OR “light emitting diode therapy” OR LEDT OR “red light therapy” OR “cold laser”) AND TS = (“glycated hemoglobin” OR HbA1c OR “fasting glucose” OR insulin OR HOMA-IR OR OGTT OR “oral glucose tolerance” OR “glycemic control” OR “inflammatory markers” OR CRP OR cytokine* OR “tumor necrosis factor” OR TNF-alpha OR interleukin* OR IL-6 OR IL-1beta OR oxidative stress OR ROS OR neuropathy OR “nerve conduction” OR “neuropathy symptom score” OR “autonomic dysfunction” OR “heart rate variability” OR neuroinflammation OR BDNF OR NGF OR “quality of life”))
Cochrane Library	#1 (“type 2 diabetes” OR T2DM OR “adult onset diabetes” OR “non-insulin dependent diabetes”):ti,ab,kw #2 (“photobiomodulation” OR “photobiomodulation therapy” OR PBMT OR “low level laser therapy” OR LLLT OR “light emitting diode therapy” OR LEDT OR “red light therapy” OR “cold laser”):ti,ab,kw #3 (“glycated hemoglobin” OR HbA1c OR “fasting glucose” OR insulin OR HOMA-IR OR OGTT OR “oral glucose tolerance” OR “glycemic control”):ti,ab,kw #4 (“inflammatory markers” OR CRP OR cytokine* OR “tumor necrosis factor” OR TNF-alpha OR interleukin* OR IL-6 OR IL-1beta OR oxidative stress OR ROS):ti,ab,kw #5 (neuropathy OR “nerve conduction” OR “neuropathy symptom score” OR “autonomic dysfunction” OR “heart rate variability” OR neuroinflammation OR BDNF OR NGF OR “quality of life”):ti,ab,kw #6 #3 OR #4 OR #5 #7 #1 AND #2 AND #6
CINAHL	(MH “Diabetes Mellitus, Type 2+” OR “type 2 diabetes” OR T2DM OR “adult onset diabetes” OR “non insulin dependent diabetes”) AND (MH “Laser Therapy, Low Level+” OR MH “Phototherapy+” OR “photobiomodulation” OR “photobiomodulation therapy” OR PBMT OR “low level laser therapy” OR LLLT OR “light emitting diode therapy” OR LEDT OR “red light therapy” OR “cold laser”) AND ((“glycated hemoglobin” OR HbA1c OR “fasting glucose” OR insulin OR HOMA-IR OR OGTT OR “oral glucose tolerance” OR “glycemic control”) OR (“inflammatory markers” OR CRP OR cytokine* OR “tumor necrosis factor” OR TNF-alpha OR interleukin* OR IL-6 OR IL-1beta OR oxidative stress OR ROS) OR (“neuropathy” OR “nerve conduction” OR “neuropathy symptom score” OR “autonomic dysfunction” OR “heart rate variability” OR neuroinflammation OR BDNF OR NGF OR “quality of life”))

Note: The asterisk (*) indicates truncation to retrieve word variants in the database search.
